# Differences in Simulated Refractive Outcomes of Photorefractive Keratectomy (PRK) and Laser In-Situ Keratomileusis (LASIK) for Myopia in Same-Eye Virtual Trials

**DOI:** 10.3390/ijerph17010287

**Published:** 2019-12-31

**Authors:** Ibrahim Seven, Joshua S. Lloyd, William J. Dupps

**Affiliations:** 1 Cleveland Clinic, Cole Eye Institute, Cleveland, OH 44195, USA; Ibrhmsvn@gmail.com; 2 OptoQuest, Inc., Cleveland, OH 44106, USA; jlloyd@optoquest.net; 3 Department of Biomedical Engineering, Cleveland Clinic, Cole Eye Institute, Cleveland, OH 44195, USA

**Keywords:** cornea, refractive surgery, finite element analysis, laser in situ keratomileusis (LASIK), photorefractive keratectomy (PRK)

## Abstract

The use of computational mechanics for assessing the structural and optical consequences of corneal refractive procedures is increasing. In practice, surgeons who elect to perform PRK rather than LASIK must often reduce the programmed refractive treatment magnitude to avoid overcorrection of myopia. Building on a recent clinical validation study of finite element analysis (FEA)-based predictions of LASIK outcomes, this study compares predicted responses in the validated LASIK cases to theoretical PRK treatments for the same refractive error. Simulations in 20 eyes demonstrated that PRK resulted in a mean overcorrection of 0.17 ± 0.10 D relative to LASIK and that the magnitude of overcorrection increased as a function of attempted correction. This difference in correction closely matched (within 0.06 ± 0.03 D) observed differences in PRK and LASIK from a historical nomogram incorporating thousands of cases. The surgically induced corneal strain was higher in LASIK than PRK and resulted in more forward displacement of the central stroma and, consequently, less relative flattening in LASIK. This FE model provides structural confirmation of a mechanism of action for the difference in refractive outcomes of these two keratorefractive techniques, and the results were in agreement with empirical clinical data.

## 1. Introduction

The cornea is an avascular and transparent tissue that is also the principal determinant of refraction in the human eye [1]. Due to the robust link between cornea’s geometry and its refractive performance, it is the primary target for shape-altering photorefractive surgeries that treat visual disorders such as myopia, hyperopia and astigmatism. While laser refractive surgery systems afford a high level of surgical precision, the biomechanical response of the cornea to the photoablation is not explicitly accounted for in the surgical planning processes [2,3] and is an essential source of deviation of actual clinical results from theoretical expectations [4].

Structural analysis using the FEA method is a useful tool for investigating clinical hypotheses related to the biomechanical impact of surgical interventions in the eye. With recent developments in imaging technology, three-dimensional corneal tomographic datasets are readily available in a clinical setting. A combination of FEA with high precision corneal refractive surgery interventions provide a privileged clinical system in which to explore the potential of simulation-based engineering science to facilitate outcome prediction and clinical decision-making.

In a prior study [5], we investigated the predictive accuracy of a computational modeling approach to laser in-situ keratomileusis (LASIK), the most commonly performed form of keratorefractive surgery worldwide. LASIK involves the creation of a partial-thickness corneal flap that is hinged to allow access to the underlying residual stromal bed. The residual corneal stromal bed is then accessed by lifting the flap, and excimer laser ablation is performed with a spatial profile specific to the patient’s refractive error. Photorefractive keratectomy (PRK) is similar to LASIK but foregoes the flap in favor of more superficial corneal ablation. In clinical practice, PRK is often preferred in patients with thinner or atypical corneal geometries because it is structurally more conservative while achieving similar refractive outcomes to LASIK. However, surgical planning often requires programming a surgeon-specified treatment offset to account for a clinical trend toward overcorrection of myopia with PRK. The magnitude of this correction is based on historical trends with minimal patient-specific input.

In this study, we perform a same-eye trial comparing simulated corneal shape changes using a previously validated virtual LASIK cohort to simulated outcomes for PRK with the same attempted refractive change.

## 2. Materials and Methods

### 2.1. Subjects

The charts of 20 eyes of 12 subjects that underwent LASIK for myopia or myopic astigmatism were included in the study. Patients were selected by a retrospective review of corneal tomography under an institutional review board (IRB)-approved research protocol (Cleveland Clinic IRB protocol #13-213). Eyes with at least 10 mm of corneal coverage in Scheimpflug-based anterior segment scans (Pentacam HR, Oculus Optikgerate GmbH, Wetzlar, Germany) in preoperative and 3-month postoperative measurements were selected.

### 2.2. Geometry and Material Model

**Figure 1:**
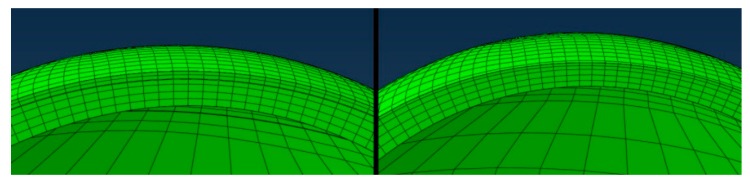
A representative mesh of preoperative corneal models for LASIK (left frame) and PRK (right frame). Both models consist of an anterior epithelial layer and underlying stromal layers. Sublayers, defined from anterior to posterior, for LASIK (**left**) are the epithelium, stromal component of the flap, flap interface wound (bolded boundary), and residual stromal bed. Sublayers for PRK (**right**) are the epithelium and residual stromal bed (**right**).

Patient-specific corneal tomographies were exported, and preoperative Cartesian coordinates for the anterior and posterior corneal surfaces were interpolated using 8th order Zernike polynomials [6]. The mean Zernike fitting error was 1.10 ± 0.19 μm. The geometries were meshed with 8-node 3D hexahedral brick elements with custom software (SpecifEye v0.9, OptoQuest, Cleveland, OH, USA). The corneal mesh was divided into 4 and 2 distinct layers for LASIK and PRK, respectively. The LASIK model consisted of epithelium, anterior stroma incorporated into the flap, the flap-residual stroma interface wound and residual stroma, while the PRK model included only epithelium and residual stroma (Figure 1). The mesh consists of 6 layers in both LASIK and PRK models. The cornea and sclera were each meshed using 6750 elements. The number of elements in the axial direction was selected based on a mesh convergence analysis. A 6-element mesh was selected based on sensitivity analysis to optimize the computational duration, summarized in Table 1, in which changes in maximum principal strain and displacement values were less than 1.0%. The sensitivity analysis reduces the likelihood that differences between PRK and LASIK will arise merely from mesh idiosyncrasies. The epithelium, sclera, and limbus were modeled as hyperelastic incompressible neo-Hookean material using W_matrix_ = c(*I*_1_–3), where *I*_1_ is the first deviatoric invariant of the left Cauchy–Green deformation tensor and W is the strain energy density function. The value of c for the epithelium was set to 0.002 megapascals (MPa), 5% of the corresponding value for stroma (see below), to reflect the negligible contribution of the epithelium to the mechanical behavior of the cornea while still allowing model convergence. The value of c was set to 0.16 and 0.2 MPa for limbus and sclera, respectively, both of which were represented as isotropic materials. The scleral value was based on a scleral shear modulus value from experimental data [7] and scaled down for the limbus. This assumption served to acknowledge the limbus as a distinct mechanical region while avoiding a material stiffness discontinuity between cornea and sclera. The stroma was represented as a nonlinear, anisotropic, hyperelastic, nearly incompressible material with depth-dependent material properties [5]. A microstructural fibril-reinforced material model representing collagen fibrils was combined with an isotropic Neo-Hookean solid extrafibrillar matrix to produce a composite corneal stroma. The cornea fibrils were represented as linear helical springs, and crimping-uncrimping behavior of these fibrils was reflected using an algorithm given by Freed et al. [8,9,10]. This equation was combined with a probability density function reported by Pinsky et al. [11] to demonstrate fibrils’ distribution within the cornea based on Meek’s [12] X-ray scatter findings. Like most biological soft tissues, the cornea exhibits some viscoelastic behaviors but is modeled here, as in prior studies [5,12], with hyper-elastic material properties.

The stromal material model equations are summarized here. The strain energy density function in the stroma is defined as:

$$W_{stroma}=W_{matrix}+\frac{1}{\pi }\int_{\;0}^{\pi }\Phi \left(r,\theta ,\varphi \right)W_{collagen}d\theta$$

$$W_{collagen}=\frac{1}{\prod }\int_{0}^{\prod }\Phi \left(R_{cornea},\varphi ;\theta \right)W_{fibril}\left(R,\varphi ;\theta \right)d\theta$$

$$\Phi \left(R_{cornea},\varphi ;\theta \right)={cos}^{2n}(\theta )+{sin}^{2n}(\theta )+c_{1}\;\;\;0\;<\;R_{\mathrm{cornea}}\;\leq \;4.5\;\mathrm{mm}$$

$$\Phi \left(R_{cornea},\varphi ;\theta \right)={sin}^{2n}(\theta -\varphi )+c_{2}\;\;\;4.5\;\mathrm{mm}\;<\;R_{\mathrm{cornea}}\;<\;5.5\;\mathrm{mm}$$

*c*_1_ = 0.45

*c*_2_ = 0.72

within the stroma, where α represents the unit sphere, W represents the strain density function, ω is the fibril distribution function, and R_cornea_ is the distance from the corneal apex. The stromal matrix was modeled as a neo-Hookean material with the material constant was depth variant. The constant c was set to 0.04 for the anterior stroma and was linearly decreased to 0.02 for the posterior stroma.

Cauchy stress of each collagen fiber is given by:

$$\sigma_{collagen}=\frac{1}{J\lambda }\frac{\delta W_{collagen}}{\delta \lambda }b\left(r,\theta ,\varphi \right)⊗b\left(r,\theta ,\varphi \right)$$

*J* = detF

where *I*_3_ is the third deviatoric Cauchy strain invariant, *b* is the fibril orientation vector, and *λ* is fiber stretch. We used an algorithm proposed by Freed et al. to calculate the collagen fiber stress based on a three-dimensional crimped helical fiber assumption, and this algorithm can be found in Appendix A of that publication [8]. The three parameters of the helical collagen fiber are the initial normalized wavelength of the crimp (set to 30.5), the initial normalized amplitude of the crimp (set to 1.51), and the elastic modulus of the collagen fibril in the linear region, which was set to 32 MPa. These normative constants were computed using a MATLAB (Mathworks, MA, USA) simulated annealing optimization algorithm based on a series of inverse FE analyses to replicate reported corneal inflation and tensile experiment outcomes, and these normative constants were assumed to be invariant across all eyes [5,9]. This material model was implemented in Abaqus using a UMAT subroutine. During our material property optimization, a cornea-only model with a small scleral rim was used to replicate the inflation experiment. Fixed boundary conditions were applied at the scleral rim to simulate the anterior inflation chamber. When performing the surgical simulations, a generic circular sclera with a 24 mm outer diameter was implemented to enable limbal node displacements, and a physiological (15 mmHg) intraocular pressure (IOP) was applied. Fixed boundary conditions were applied at the posterior pole of the sclera. No other fixture was imposed on the model as a boundary condition. 

### 2.3. Surgical Simulation

The mesh was generated using the eye’s loaded state. The wavefront-optimized ablation was modeled by changing the thickness of the cornea according to the eye-specific treatment parameters. The equations to compute wavefront-optimized (WO) ablation profile given by Mrochen et al. [13]. were modified by our group, and the geometric alteration from this photoablation was imposed on the patient-specific mesh in the loaded state [5]. Following the ablation, the unloaded state (stress-free) was computed to account for the pre-existing strain at the time of the tomography. Stress-free configurations were determined using an iterative approach described previously [14] with modifications also described previously [5]. The disruption of the fibrils during the flap creation was simulated by removing the fibril component from the strain energy equation in the flap region. A wound layer with 10 um thickness was included at the flap boundaries to simulate the convalescent (post-healing) postoperative state. The wound layer was simulated by reducing its shear and fibril modulus to a negligible value. PRK models for the same eye implemented the same ablation profile but directly below the epithelium, and fibril structure was preserved in the residual stromal bed (RSB) [15]. Finite element solutions were obtained using Abaqus v6.12 (Dassault Systemes Simulia Corp., Providence, RI, USA).

### 2.4. Corneal Curvature and Strain Analyses

After the simulation, anterior corneal surface coordinates from pre- and post-treatment geometries were exported from the solver into SpecifEye where simulated keratometry values (in diopters, D), representing the steep and flat principle axes of the corneal surface, were calculated then averaged to estimate the spherical refractive power of the cornea. Additionally, average corneal maximum principal strain (MPS) of the corneal RSB was calculated based on the circular, central 5 mm region of the pre- and post-treatment states. This strain variable has been proposed as a structural risk measure due to its high correlation to clinical risk as demonstrated in a previous large-scale computational analysis by our group [10]. The surgically induced change in the average MPS was presented to demonstrate the change in corneal mechanical response to the same IOP following a photo-ablative surgery. Pairwise (PRK vs. LASIK) changes in anterior corneal curvature and average MPS for each procedure were calculated and compared with a paired Student’s *t*-test for non-independent samples (with *p* < 0.05 indicating statistical significance). Historical changes in manifest refractive error (MRx) were obtained for each PRK and LASIK case from a regression-based nomogram software package using a global database feature that incorporates thousands of outcomes (IBRA, Zubisoft, Switzerland). Subject age was fixed at 40 for all historical outcome queries to represent the mean subject age in the study. Since corneal material properties were not adjusted for age in these simulations and because the differential effect of PRK and LASIK in identical eyes was of primary interest, keeping age constant in the nomogram entries was important to avoid asymmetrically introducing a confounding variable. The spherical equivalent (SE) of the nomogram-predicted change in MRx was calculated for PRK and LASIK. The SE refractive error values were transposed from the spectacle plane to the corneal plane to make them directly comparable to the corneal curvature results. The relationship between the magnitude of the PRK-LASIK difference and the level of attempted myopic correction was assessed by linear regression for both simulation- and nomogram-predicted results. 

## 3. Results

The mean patient age averaged across eyes was 39.3 ± 12.7 years. Clinical tomographic characteristics of the simulated eyes included a mean maximum curvature (maximum K) of 44.12 ± 1.21 D (mean ± SD), mean apical corneal thickness of 570.5 ± 21.9 um, and mean minimum corneal thickness of 568.1 ± 21.7 um. 

**Figure 2:**
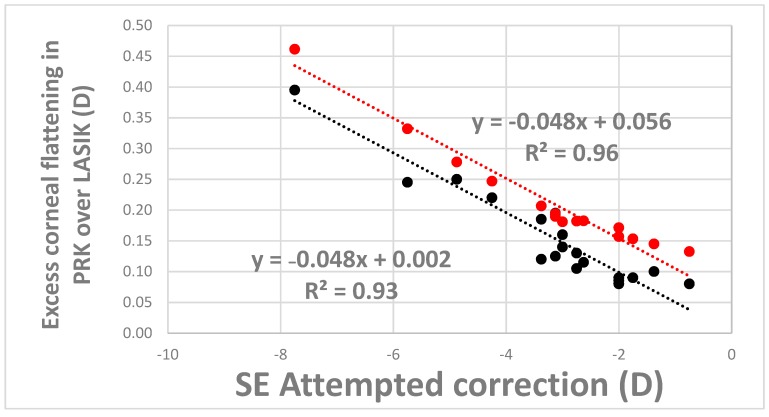
Additional corneal flattening observed in PRK over those observed in LASIK from simulations (black group), and global historical nomogram (red group) plotted as a function of attempted spherical equivalent (SE) refractive error. (D: Diopters).

The mean programmed refraction correction was −3.18 ± 1.69 D for the spherical component and −0.50 ± 0.35 D for the cylindrical component. Mean keratometry power (K_average_) decreased by an additional 0.17 ± 0.10 D in PRK simulations compared to LASIK (*p* < 0.001). Table 2 shows individual differences in K_1_, K_2_, K_average_ and average MPS between LASIK and PRK simulations. In both simulation- and nomogram-predicted outcomes, the magnitude of relative PRK overcorrection increased as attempted correction increased. Ninety-three percent and 96% of the variance of the PRK overcorrection was accounted for by a linear relationship based on simulation- and nomogram-predicted outcomes, respectively. Figure 2 demonstrates these linear relationships for simulation- (0.002D − (0.048D × Δattempted spherical equivalent correction), black line, *p* < 0.001) and nomogram-predicted (0.056D − (0.048D × Δattempted spherical equivalent correction), red line, *p* < 0.001) outcomes. Historical nomogram output suggested an average 0.22 ± 0.09 D additional corneal flattening in PRK over LASIK. The average difference in flattening between simulation and nomogram predictions between PRK and LASIK was −0.054 ± 0.03 D, with identical slope terms relating the degree of overcorrection to magnitude of attempted correction.

**Figure 3:**
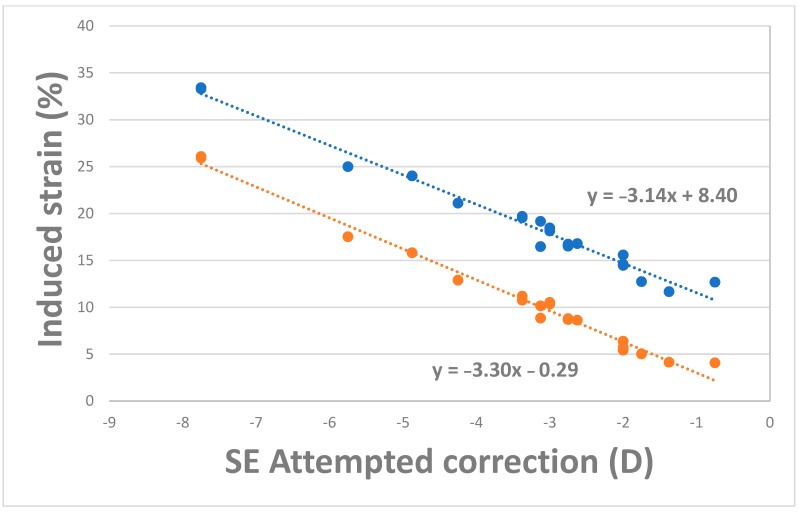
Post-surgical increase (given as a percentage) in average MPS as a function of SE of the correction for LASIK (blue) and PRK (orange), SE: spherical equivalent (diopters), MPS: Maximum principal strain.

**Figure 4:**
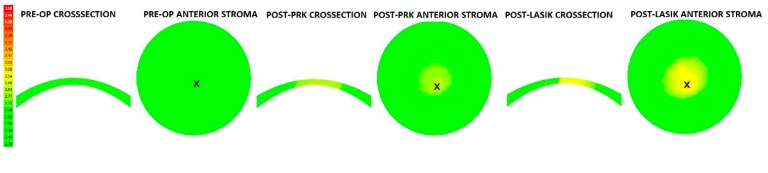
Example of maximum principal strain (MPS) maps for full-thickness vertical cross-sections and *en face* anterior stromal regions for pre-operative, post-PRK, and post-LASIK simulations, respectively. Strain maxima are indicated by an ‘x’ on the map. Higher MPS values were incurred in LASIK than in PRK in all cases.

Figure 3 shows surgically induced percentage strain increases for PRK and LASIK cases. PRK consistently induced less strain in the residual stromal bed than LASIK. The surgically induced percentage strain increased as attempted correction increased (Figure 3). In total, 97.67% and 90.00% of the variances were accounted for by a linear relationship for LASIK and PRK, respectively. The differences in induced strain formed an offset when linear fits were introduced for both LASIK and PRK groups (Figure 3). Figure 4 demonstrates MPS maps vertical meridian cross-section of full-thickness stroma and *en face* anterior stroma for PRK and LASIK with the same strain scale.

**Figure 5:**
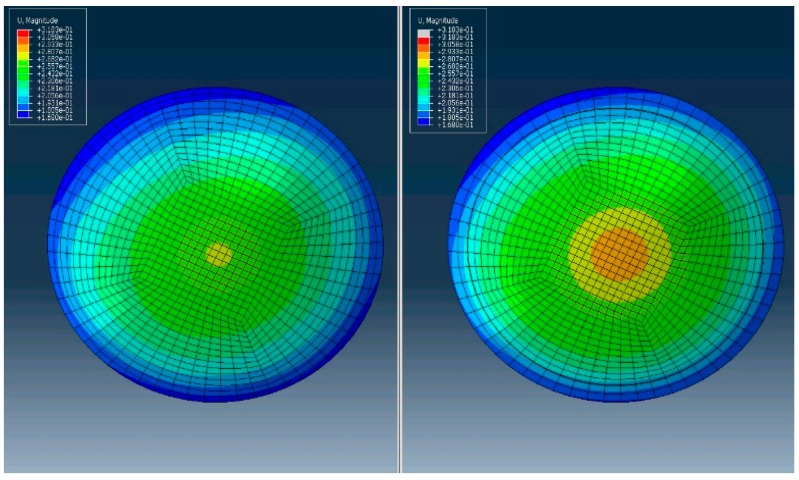
Magnitude of the displacements (mm) in three principal directions (x, y, z) for post-PRK (**left**) and post-LASIK models (**right**) with identical color scales. Residual stromal bed displacements were greater in LASIK than in PRK.

**Figure 6:**
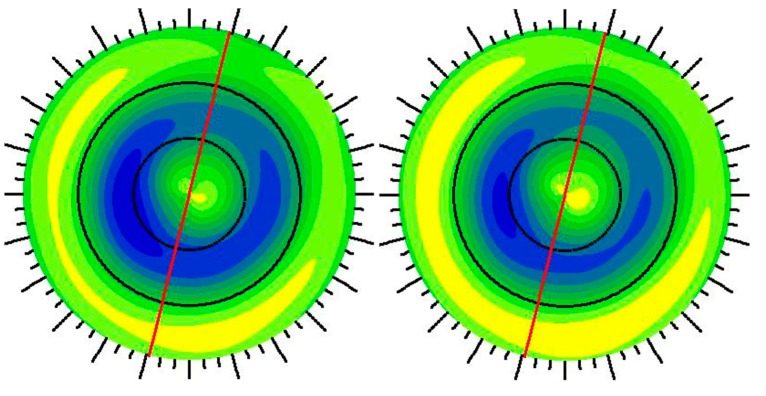
Axial corneal curvature maps (Diopters) for post-PRK (**left**) and post-LASIK models (**right**).

Higher displacements in response to a physiologic IOP were observed in post-LASIK than post-PRK corneal models. Displacement maps for post-PRK (left) and post-LASIK (right) models are compared in Figure 5. Axial curvatures for the same post-PRK (left) and post-LASIK (right) models are demonstrated in Figure 6.

## 4. Discussion

LASIK is one of the world’s most commonly performed procedures and is associated with high levels of patient satisfaction [16]. PRK is often preferred as an alternative to LASIK for patients with thinner corneas or atypical curvature features. The underlying reason for this preference is the clinical perception that PRK conserves more of the biomechanical strength of the cornea by foregoing the flap, and this is generally supported by the relative rarity of post-surgical corneal ectasia after PRK.

In this study, we compared the induced biomechanical changes associated with these two techniques and their resulting visual outcomes for a same amount of attempted refractive correction using a previously validated finite element model from a clinical myopic LASIK series. The model incorporates patient-specific 3-D corneal geometry, case-specific treatment geometry and experimental evidence-based corneal anisotropy and depth-dependent material property assumptions to more realistically simulate the corneal optomechanical response. While previous studies investigated LASIK [5] and PRK [15] simulations alone using different material model approaches, and our group published a comparison between flap-based and flapless lenticule extraction techniques [17], to our knowledge no study has directly compared the biomechanical and optical responses of PRK and LASIK.

This series of virtual subjects demonstrated a tendency toward greater corneal flattening in PRK simulations than in LASIK simulations with equivalent patient-specific geometries and subject-invariant material property assumptions. This finding is consistent with a clinical need for surgeon adjustments to the treatment algorithm to avoid unintended hyperopic refractive error when PRK is selected instead of LASIK. The surgically induced strain following PRK also was consistently less than LASIK. The difference in strain did not significantly change as the correction amount changed. The most important differential factor for the strain difference was the added biomechanical impact of LASIK flap creation. This difference existed for the small corrections as well. However, the combination of ablation and flap induced strains produced more than 30% strain change for our cases that underwent the highest attempted correction (SE = −7.75 D). Our prior work serves as a precedent for using the magnitude of the maximum principal strain as a structural risk variable in the context of laser refractive surgery [9,17], and several other examples of its use exist for quantifying structural risk [18,19].

Differences in wound healing following LASIK and PRK are not fully accounted for in these comparisons apart from the fact that a nominal wound layer was included at the LASIK flap interface in LASIK simulations and no such interface was present in the PRK simulations. The LASIK simulations were compared to 3-month postoperative geometries in the previous validation work, which is a commonly accepted geometric stability endpoint for LASIK. Modifications of stromal biomechanical properties related to wound healing after PRK are a possible source of error in this comparison and are not explicitly considered here. Furthermore, we do not account for creep or other viscoelastic phenomena that may occur in the postoperative period, and the comparison makes the implicit assumption that any difference in these effects in the first 3 months after LASIK and PRK are negligible. While the corneal material model treats the cornea as anisotropic, the sclera and limbus are represented by simplified isotropic material properties to facilitate computational efficiency. Also, while differences in ablative efficiency between PRK and LASIK due to intraoperative hydration differences have been proposed as an explanation for this phenomenon, the present study demonstrates differences that closely match historical differences without appealing to hydration effects. The current work suggests that differences in residual stromal bed strains result in greater relative corneal steepening in LASIK that undermines myopic correction. This supports the notion that PRK has less tendency to weaken the central cornea than LASIK in myopic surgery. 

This study did not account for epithelial thickness profile changes following LASIK and PRK. Central corneal epithelial thickness tends to increase following a myopic ablation, and peripheral epithelial thickness decreases according to the degree of curvature change produced by LASIK or PRK [20]. The hypothesis for this phenomenon is that the epithelium profile changes as a result of hypertrophy and thinning in response to the mechanical interaction between the eyelids and regional differences in corneal elevation. Both PRK and LASIK exhibit these effects and we don’t expect the difference in refractive effects between LASIK and PRK to be confounded since the same ablation profile was used for each case. The material property assumptions for cornea and sclera are generalized and do not account for patient-specific differences that could impact the comparison of LASIK and PRK.

## 5. Conclusions

In conclusion, these findings offer insight into the biomechanical differences between PRK and LASIK, the refractive consequences of these differences and the potential utility of patient and case-specific approaches to surgical planning.

## Figures and Tables

**Table 1:** Mesh convergence analysis. Δstrain%: Percentage change in the highest maximum principal strain, ΔU%: percentage change in highest maximum displacement.

Layers	Cornea Elements	ΔStrain%	ΔU%
4	4500	2.93	0.38
5	5625	1.42	0.13
6	6750	1.05	0.08
7	7875	0.70	0.00
8	9000	0.34	0.00

**Table 2:** The differences between post-PRK and post LASIK in K_steep_: simulated keratometry steep principle axis, K_flat_: simulated keratometry flat principle axis, Kaverage: average simulated keratometry, MPS: maximum principal strain in response to the corresponding photoablation attempted correction. (D: diopters, sph: spherical, cyl: cylinder, se: spherical equivalent, SE: spherical equivalent).

	Attempted Correction			Curvature and Strain			
Patient	SPH (D)	CYL (D)	SE (D)	Ksteep (D)	Kflat (D)	Kaverage (D)	ΔMPS (%)
1	−4.00	−0.50	−4.25	0.22	0.22	0.22	8.22
2	−3.00	−0.25	−3.13	0.20	0.19	0.19	7.62
3	0.00	−1.50	−0.75	0.08	0.08	0.08	8.59
4	−3.00	−0.75	−3.38	0.11	0.13	0.12	8.94
5	−2.75	−0.75	−3.13	0.12	0.13	0.13	9.02
6	−1.75	−0.50	−2.00	0.07	0.10	0.09	8.08
7	−2.50	−0.50	−2.75	0.10	0.11	0.11	7.82
8	−7.50	−0.50	−7.75	0.40	0.39	0.40	7.37
9	−7.50	−0.50	−7.75	0.39	0.40	0.40	7.33
10	−2.00	0.00	−2.00	0.07	0.09	0.08	9.85
11	−2.25	−0.75	−2.63	0.11	0.12	0.12	8.17
12	−2.50	−0.50	−2.75	0.13	0.13	0.13	7.95
13	−5.50	−0.50	−5.75	0.24	0.25	0.25	7.47
14	−2.50	−1.00	−3.00	0.14	0.14	0.14	8.10
15	−2.50	−1.00	−3.00	0.15	0.17	0.16	7.62
16	−2.00	0.00	−2.00	0.08	0.10	0.09	9.14
17	−4.25	−1.25	−4.88	0.25	0.25	0.25	8.19
18	−2.75	−1.25	−3.38	0.18	0.19	0.19	8.37
19	−1.75	0.00	−1.75	0.09	0.09	0.09	7.71
20	−1.25	−0.25	−1.38	0.10	0.10	0.10	7.53
			Mean	0.16 ± 0.10	0.17 ± 0.09	0.17 ± 0.10	8.15 ± 0.67
			*p*	<0.0001			
